# Is raloxifene associated with lower risk of mortality in postmenopausal women with vertebral fractures after vertebroplasty?: a hospital-based analysis

**DOI:** 10.1186/s12891-015-0670-7

**Published:** 2015-08-19

**Authors:** Fu-Mei Su, Ying-Chou Chen, Tien-Tsai Cheng, Wei-Che Lin, Chun-Chung Lui

**Affiliations:** Department of Rheumatology, Kaohsiung Chang Gung Memorial Hospital, Chang Gung University College of Medicine, 123 Ta-Pei Road, Niao-Sung District Kaohsiung, 833 Taiwan; Department of Radiology, Kaohsiung Chang Gung Memorial Hospital, Chang Gung University College of Medicine, Kaohsiung, 833 Taiwan

## Abstract

**Background:**

Osteoporotic fractures are associated with mortality in postmenopausal woman. Whether raloxifen treatment after vertebroplasty can reduce mortality is unclear in this group. To compare the effect of raloxifene and no osteoporosis treatment on the risk of mortality after vertebroplasty, we designed this study.

**Methods:**

This was a retrospective study (January 2001 to December 2007). Follow-up for each participant was calculated as the time from inclusion in the study to the time of death, or to December 31^st^, 2013, whichever occurred first. All of the patients underwent baseline bone density studies, and age and body mass index (kg/m^2^) were recorded. All associated medical diseases such as diabetes, hypertension, and liver and renal disease were recorded.

**Results:**

One hundred and forty-nine patients with vertebral fractures were enrolled, of whom 51 used raloxifene and 98 patients did not receive any anti-osteoporotic therapy. At the end of the follow-up period, 62 patients had died and 87 were still alive. The treated patients had a lower mortality rate than those who did not receive treatment (*P* = 0.001, HR = 3.845, 95 % CI 1.884-7.845). The most common cause of mortality was sepsis, and those who received raloxifene had a lower rate of sepsis compared to those who did not receive treatment (*P* < 0.001).

**Conclusions:**

Effective treatment with raloxifene may had a lower mortality rate in patients with postmenopausal osteoporosis-related vertebral fractures after vertebroplasty.

## Background

Raloxifene is a selective estrogen-receptor modulator that has been shown to prevent bone loss. In postmenopausal women with osteoporosis, treatment with raloxifene has been shown to decrease markers of bone turnover by 30 to 40 % after one year of usage, and increase bone density at several scanning sites by 2 to 3 % after three years of use [1–3]. Raloxifene has also been shown to decrease the incidence of vertebral fractures by 30 to 50 %, depending on dosage, but not the incidence of hip fractures or other non-vertebral fractures [1].

Osteoporotic fractures of the spine are common with aging, and the lifetime risk of a symptomatic vertebral compression fracture has been estimated to be 18 % for women and 11 % for men [4]. In addition, painful, clinically apparent vertebral fractures have been reported to increase overall mortality by up to 15 % [4, 5]. Furthermore, some individuals will become disabled by severe pain that lasts for longer than 2 to 3 months.

Data from studies on raloxifene used to reduce the risk of fractures can provide a way to test the hypothesis that treating osteoporosis reduces the risk of death. If treating osteoporosis does have a positive impact on mortality, there would be several important potential implications for managing skeletal health specifically, and for health care for women in general. Therefore, in this study, we investigated whether raloxifene treatment affects mortality rates in postmenopausal woman with vertebral fractures.

## Methods

This was a retrospective review of osteoporosis patients with vertebral fractures. The Institutional Review Board of Kaohsiung Chang Gang Memorial Hospital approved this study, and it was conducted in accordance with the Declaration of Helsinki and the International Conference on Harmonization of Good Clinical Practice Guidelines. According to Taiwan law, no additional informed consent was required, and patient information was anonymized and de-identified before data analysis.

The inclusion criteria were postmenopausal women with a radiological diagnosis of a vertebral fracture followed by a vertebral augmentation procedure for a painful vertebral compression fracture, and failure of conservative pain management. All patients were examined with magnetic resonance imaging, and we obtained the electronic medical records of all patients who underwent vertebral augmentation procedures. Patients were excluded if their fracture had a pathological source or had been caused by more than minimal trauma. We choose those with raloxifen treatment (60 mg/d after vertebroplasy) and those without treatment with any anti-osteoporotic therapy for comparison.

Follow-up for each participant was calculated as the time from inclusion in the study to the time of death, or to December 31^st^, 2013, whichever occurred first. All of the patients underwent baseline bone density studies, and age and body mass index (kg/m^2^) were recorded. All associated medical diseases such as diabetes, hypertension, and liver and renal disease were recorded.

### Statistical analysis

Statistical analysis was performed using SPSS software, version 21.0 (SPSS, Chicago, IL, USA). Kaplan-Meyer analysis with the log rank test was performed for different groups. Comparisons between independent means were analyzed using the independent t test, and relationships between categorical variables were evaluated by the chi-square test. Cox regression analysis was used to adjust for potential confounding factors. A *P* value of less than 0.05 was considered to be statistically significant.

## Results

One hundred and forty-nine patients with vertebral fractures were enrolled in this study, of whom 51 used raloxifene and 98 did not receive anti-osteoporotic therapy. All were grade 3 by semiquantitative grading scale for vertebral fracture and T score < -2.5 with bone densitometry. The mean age at the index day was 74.24 ± 7.62 years in the raloxifene group and 72.52 ± 9.70 years in those who did not receive treatment. The mean follow-up period was 7.08 ± 3.67 years.

At the end of the follow-up period, 62 patients had died and 87 were still alive. The patients who received raloxifene survived for longer than those who did not receive treatment (*P* = 0.04). There were no significant differences in body mass index, number of vertebral fractural, previous hip fracture,or underlying diseases including diabetes, hypertension, cardiovascular disease, pulmonary disease, liver disease, or neurological diseases (Table 1).

**Table 1:** Baseline characteristics of the study patients

		Raloxifene	No treatment	*P* value
Age (years)		74.24 ± 7.62	72.52 ± 9.70	0.238
Body mass index (kg/m^2^)		22.80 ± 4.82	24.12 ± 4.85	0.116
Survival years		8.06 ± 2.63	6.11 ± 4.33	0.004
Fracture no (spine)		1.83 ± 1.07	1.48 ± 1.48	0.158
Previous hip fracture	No	46(90.2 %)	88(89.8 %)	0.592
	Yes	5(9.8 %)	10(10.2 %)	
Diabetes	No	38(74.5 %)	73(74.5 %)	0.581
	Yes	13(25.5 %)	25(25.5 %)	
Hypertension	No	25(49.0 %)	46(46.9 %)	0.864
	Yes	26(51.0 %)	52(53.1 %)	
Neurological disease	No	50(98.0 %)	97(99.0 %)	1
	Yes	1(2.0 %)	1(1.0 %)	
Pulmonary disease	No	49(96.1 %)	92(93.9 %)	0.716
	Yes	2(3.9 %)	6(6.1 %)	
Cardiovascular disease	No	49(96.1 %)	93(94.9 %)	1
	Yes	2(3.9 %)	5(5.1 %)	
Liver disease	No	47(92.2 %)	91(92.9 %)	1
	Yes	4(7.8 %)	7(7.1 %)	

**Fig. 1:**
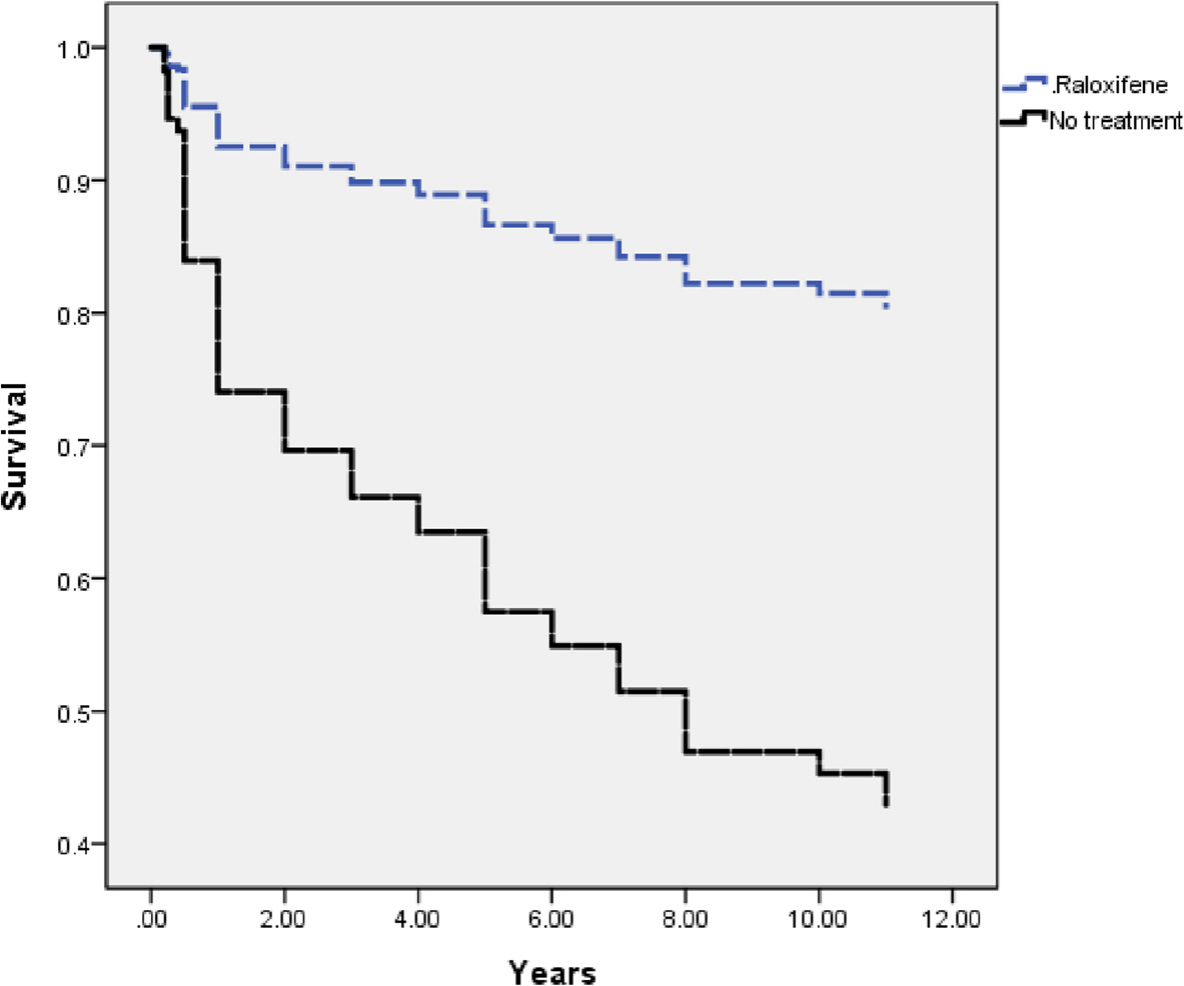
Kaplan-Meier survival curves for raloxifen therapy (dashed line) and no treatment (solid line)

**Fig. 2:**
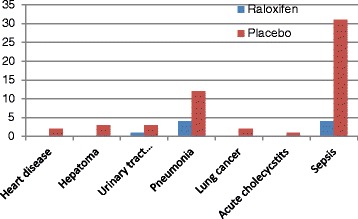
Comparison of the of the death between raloxifene and placebo group

Raloxifene therapy had a significant effect on survival according to Kaplan-Meier curves (Fig. 1). After adjusting for potential confounding factors such as diabetes, hypertension, cardiovascular disease, pulmonary disease, liver disease and neurological diseases, the patients treated with raloxifene still had a lower mortality rate than those who did not receive treatment (*P* = 0.001, HR = 3.845, 95 % CI 1.884-7.845) (Table 2). The most common cause of mortality overall was sepsis, however the rate of sepsis was significantly lower in the raloxifene group compared to the no treatment group (*P* < 0.001) (Fig. 2).

**Table 2:** Effect of raloxifene on mortality after adjusting for variables

	Regression coefficient	SE	*P* value	HR
Age (years)	.029	.018	.105	1.029
Body mass index (kg/m^2^)	-.032	.030	.289	.968
Diabetes	-.567	.294	.054	.567
Hypertension	-.159	.273	.560	.853
Neurological disease	.235	1.057	.824	1.265
Liver disease	-.554	.497	.265	.574
Cardiovascular disease	.676	.730	.355	1.966
Pulmonary disease	.214	.604	.723	1.239
Raloxifene use	1.347	.364	.000	3.845

Abbreviations: *SE*: standard error; *HR*: hazard ratio

## Discussion

Raloxifene is a non-steroidal benzothiophene analog that has been shown to inhibit the growth of estrogen-receptor-dependent dimethylbenzanthracene-induced mammary tumors and reduce the occurrence of nitrosomethylurea-induced mammary tumors in rats. It is classified as a selective estrogen-receptor modulator on the basis of studies in which it prevented bone loss and lowered serum cholesterol concentrations without stimulating the endometrium [6–9].

Theoretically, effective prevention and treatment strategies can be implemented once a high-risk individual has been identified. It is essential that evidence-based recommendations be incorporated into clinical practice [10]. Despite the availability of effective treatment in the Taiwan health care system, effective osteoporosis interventions are not optimal for women with a history of osteoporotic vertebral fractures. This is consistent with a population-based study, in which only one in five patients with a fragility fracture received treatment for osteoporosis within the following year [11]. Other studies have also reported insufficient levels of treatment, with the probability of being treated being inversely related to age and lower among older women [12–14].

Decisions regarding the treatment of postmenopausal osteoporosis should be based on the patient’s risk for fractures, drug efficacy and the side effects of these drugs. Individuals with a fragility vertebral fracture should always be treated when not contraindicated, because the risk of subsequent vertebral fractures is very high [15]. Based on the scientific evidence, raloxifene appears to be a good option.

Established osteoporosis has been associated with a high mortality rate after adjusting for age and comorbidities [16, 17], and prevalent vertebral deformities have been reported to predict increased mortality and fracture rates in both men and women [18–23]. Treatment for osteoporosis with established methods for vertebral and non-vertebral fractures has been reported to reduce mortality in older, frailer individuals with osteoporosis who are at a high risk of fractures [24–26].

In clinical practice, some patients with vertebral fractures do not receive medical therapy after vertebroplasty, and this may contribute to subsequent vertebral fractures and increased mortality [27]. Our results show that raloxifene therapy can reduce mortality, underscoring the importance of educating patients with osteoporosis about the value of raloxifene therapy. We also noted a decreased incidence of sepsis after raloxifene treatment compared to those who did not receive treatment.

It is not clear how raloxifene reduce risk of death due to infection [28]. Estrogen receptor ligands have been shown to reduce bacteremia and mortality in experimental models of infection [29]. Because vertebral fractures have been associated with increased pulmonary causes of mortality [30], so vertebral fracture might be associated with shallow respiration and increased risk of pneumonia and associated sepsis. So raloxifen reduces the risk of sepsis by decrease vertebral fracture.

A possible reason for this may be because raloxifene therapy decreases the frequency of subsequent fractures, thereby improving mobility and decreasing the infection rate.

There are several limitations to his study. First, the number of vertebral fractures was probably underestimated, given the inherent difficulty of diagnosing them. For instance, only about a third of cases of all vertebral deformities detected on radiographs receive medical attention, and less than 10 % require admission to hospital. Second, the sample size was small. Third, there was a lack of complete bone mineral density data after osteoporosis treatment. However, in this single-center cohort, we collected as much data as possible, and this study included only fragility fractures in patients older than 50 years without a secondary etiology. Thus, the patients’ fractures were due to osteoporosis.

This study also has a number of strengths. First, it was a long-term cohort study with a mean follow-up period of 7 years, which made it possible to gather sufficient follow-up data on survival. In addition, baseline magnetic resonance imaging scans were taken for each participant, all of whom had clinically diagnosed vertebral fractures. Thus, we were able to exclude other secondary causes of vertebral fractures such as cancer or pyogenic infections.

## Conclusions

In conclusion, our results showed that raloxifene therapy can reduce the mortality rate of postmenopausal women with osteoporotic vertebral fractures. After adjusting for comorbidities, the patients who received raloxifene therapy still had a lower mortality rate. Thus, optimal management with raloxifene may reduce the risk of death.
